# Large nipple areolar complex angiofibroma treated with combined surgical debulking and carbon dioxide laser therapy – a case report

**DOI:** 10.1080/23320885.2020.1724513

**Published:** 2020-02-08

**Authors:** Karen B. Lu, Anamaria Parus, Ashley Siegel, Candace Glenn, David M. Plank

**Affiliations:** a Plastic Surgery, University of Texas Medical Branch at Galveston (University of Texas Libraries), Galveston, TX, USA; b Plastic Surgery, University of Central Florida College of Medicine, Orlando, FL, USA; c Mid Florida Dermatology and Plastic Surgery, Orlando, FL, USA

**Keywords:** Fibroadenoma, nipple-areolar complex, CO_2_ laser, debulking, tuberous sclerosis, carbon dioxide laser, adenofibroma

## Abstract

Here, we describe single case review of a 14-year-old female who presented with an angiofibroma on the right nipple areolar complex, which was treated successfully with debulking and CO_2_ laser. After 8 months of follow up, there has still been no recurrence of disease.

## Introduction

Cutaneous angiofibromas are benign fibrous neoplasms and can be found on different areas of the body and the face [1]. The clinical presentation of angiofibromas may differ between patients and areas of the body. Histological examination, however, shows angiofibromas are consistently comprised of dilated capillaries in the papillary dermis and a high number of stellate and spindle shaped fibroblasts within dermal collagen [2]. Most angiofibromas, especially those of the face, are associated with tuberous sclerosis (TS). Up to 75% of individuals with TS will develop angiofibromas, but the incidence of angiofibromas in patients without TS is unknown [3]. There are limited number of cases describing fibroadenomas associated with the nipple areolar complex (NAC). One study identified 11 patients with angiofibromas of the NAC, all associated with TS [2]. Below, we present the case of a teenage female with angiofibroma of the NAC, with no associated TS, and we describe a different approach to treatment of benign NAC neoplasms.

## Case report

**Figure 1.**
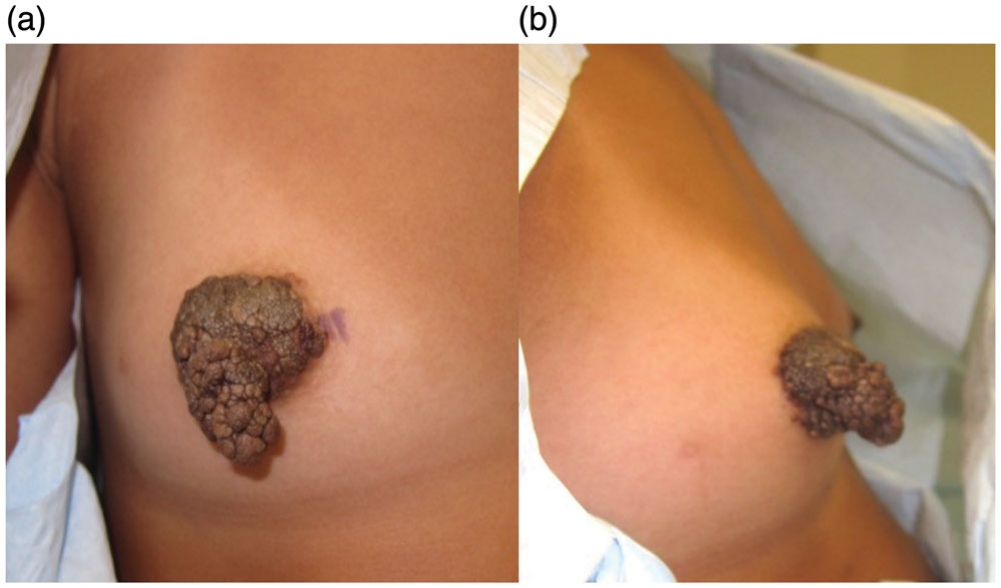
Angiofibroma right NAC. (a) Anterior view of the mass. (b) Lateral view of the mass.

**Figure 2.**
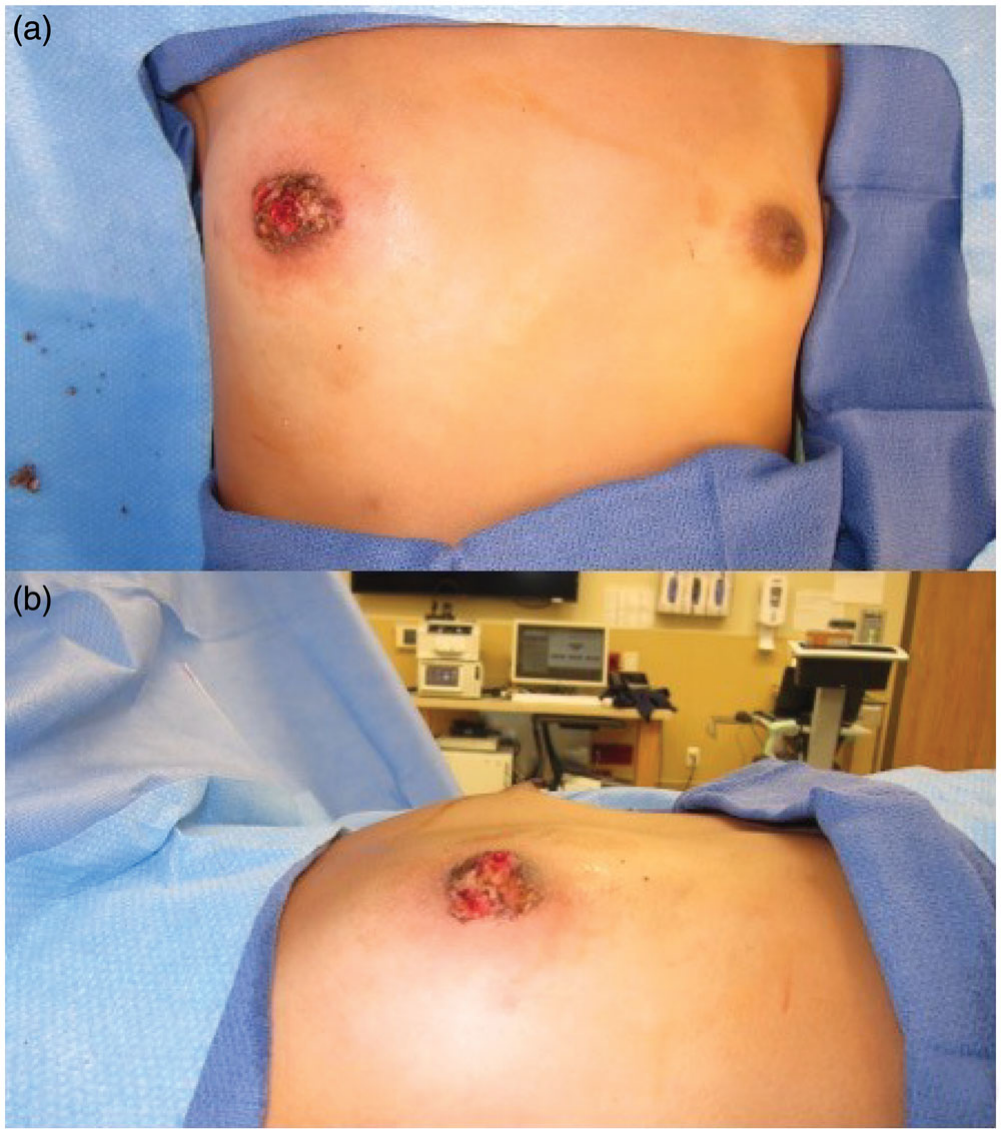
Using loop cautery, the angiofibroma was carefully, tangentially excised to the dermis of the skin. (a) Anterior view. (b) Lateral view.

**Figure 3.**
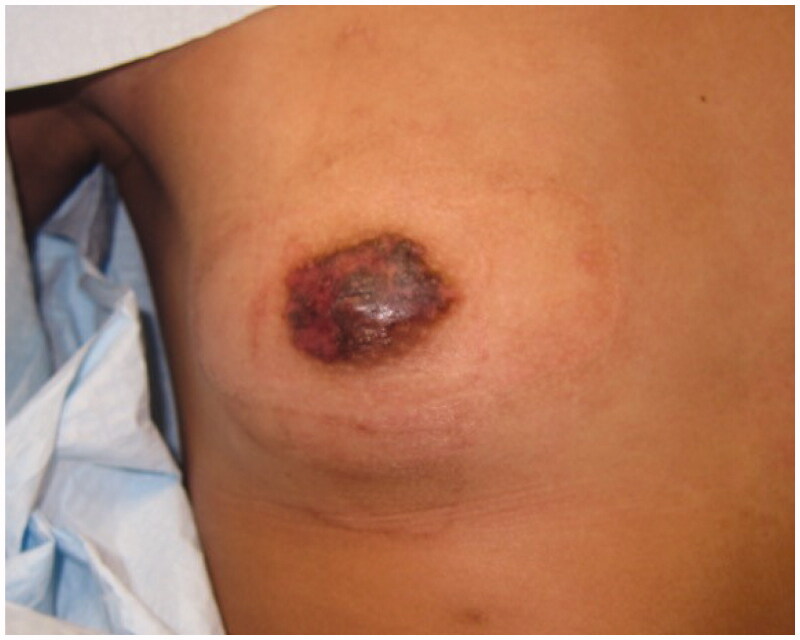
Two months following surgical debridement. Residual angiofibroma at the perimeter of the NAC underwent CO_2_ laser ablation.

**Figure 4.**
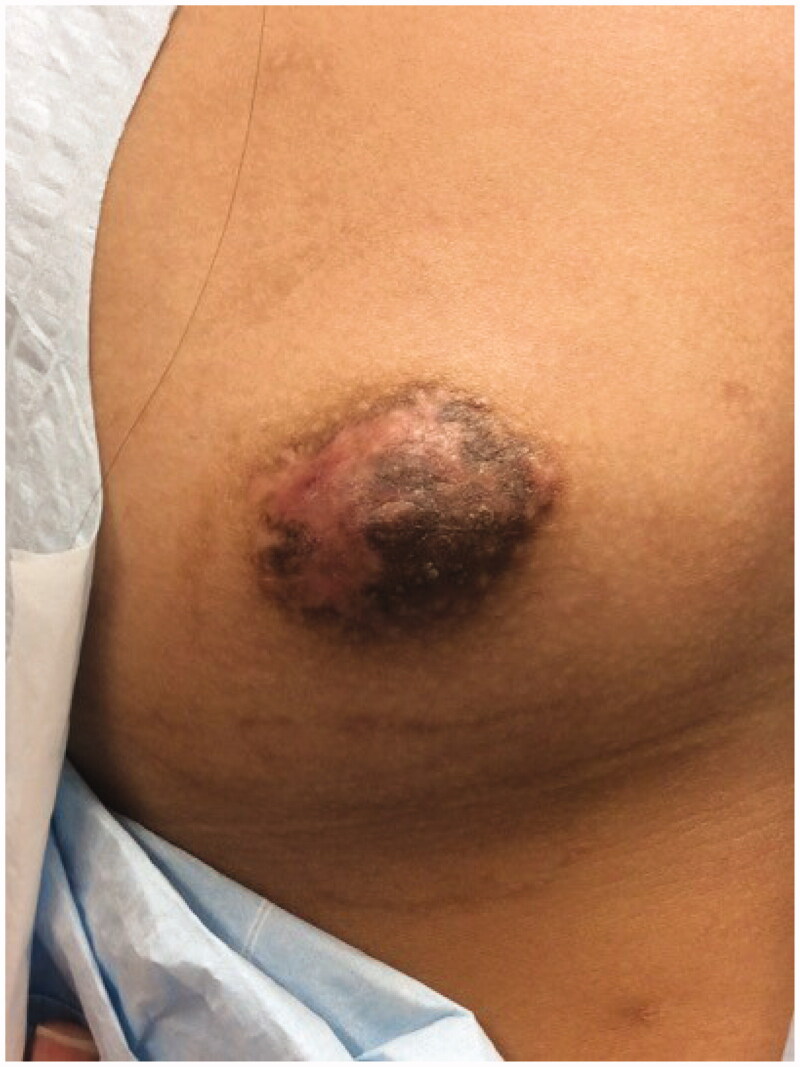
One month following second CO_2_ laser ablation of the residual NAC angiofibroma. The nipple is noted to have more definition following the second treatment. Laser treatments were separated by 4-week interval. Candela CO_2_RE. Mode: classic. Fractional density: 100%. Fluence: 2 mJ.

Informed consent for writing this case report was obtained from patient’s parents and, given the nature of this case report, IRB was not required. A 14-year-old Tanner stage 3 female presented with 2-year history of a growing exophytic, mass lesion encompassing her entire right NAC (Figure 1), without involvement of surrounding tissue. The patient stated the lesion began growing at the onset of puberty. However, she only sought medical care because of bleeding from the mass. The patient was seen by two other plastic surgeons which recommended removal of the NAC followed by reconstruction, which is the standard therapy for benign neoplasms of the NAC. The lesion was located on the right NAC and measured 3.0 cm × 3.5 cm × 3.2 cm. Multiple biopsies of the lesion were sent to pathology which demonstrated angiofibroma confirmed by two separate laboratories. Due to potential asymmetrical breast growth as a result of breast bud injury, the patient was offered a multiple modality treatment for her large NAC angiofibroma. The treatment plan consisted of tangential debulking of the mass followed by CO_2_ laser ablation of residual disease (Figure 2). Surgical debulking of the angiofibroma occurred using loop cautery. The lesion was tangentially excised to the dermis of the NAC (Figure 2). Complete removal was assessed by visual observation of the dermis. At two months after surgical debulking, patient underwent her first treatment with CO_2_ laser, which targeted residual angiofibroma at the border of the NAC (Figure 3). We chose to start laser therapy at the two-month interval to allow full epithelialization and to ensure viability of the NAC. CO_2_ laser settings were fusion at 286 J/cm^2^. The patient underwent a second treatment with CO_2_ laser after a 4-week interval (Figure 4) using the same settings. Each treatment session, we performed one pass over the affected area of the right NAC. The CO_2_ laser sessions were done under compounded topical anesthesia to numb the NAC for treatment. Follow up at 8 months demonstrated no recurrence of disease, assessed by lack of visualization of aberrant tissue growth. In addition, the patient has continued to have normal breast development without observed asymmetry.

## Discussion

Benign neoplasms of the NAC are traditionally managed by complete removal of the NAC, followed by reconstruction. However, in an adolescent female, special consideration should be taken regarding removal of the NAC, as this can disrupt eventual breast feeding. Breast milk travels through the ducts which are released from the center of the nipple [4]. Removal of the NAC, therefore, would prevent breast feeding from the affected side after pregnancy. Due to these consequences, it is critical to proceed conservatively and preserve as much tissue as possible.

Due to these considerations, a multimodality approach with tangential debulking and CO_2_ laser ablation were recommended. Our goal was to remove as much of the lesion as possible with surgical debulking, while the CO_2_ laser therapy was used to refine and prevent recurrence. Our main goal was to preserve the NAC and minimize structural distortion of the breast. Alternative therapy for benign neoplasms of the NAC requires removal and reconstruction with increased risk for complications, loss of ability for breastfeeding, and cicatricial damage to the breast. Our adolescent patient required special consideration due to her future breast maturation. Our combined therapy of debulking followed by CO_2_ laser allows a staged approach in treatment in benign neoplasms of the NAC which the breast parenchyma is minimally affected.

Angiofibromas have been treated with surgical excision, curettage, dermabrasion, electrocautery, topical rapamycin and laser ablation [5]. The types of lasers previously described in literature for treatment of angiofibromas are CO_2_, argon, erbium:YAG and/or a dye laser [5–8]. Of these laser modalities, CO_2_ treatment has been the most widely utilized method. Ali et al.’s case series report nine subjects who underwent CO_2_ laser ablation of angiofibromas of the face, all whom of reported alleviation of the angiofibromas and would recommend the treatment to others [5]. Patients received between 1 and 17 treatments, 17 being the outlier; the majority of patients received less than five treatments.

An earlier study by Papadavid et al. showed 10 out of 13 patients with fibrous or protuberant angiofibromas treated with a single session of CO_2_ laser ablation had considerable improvement [9]. However, persistent hypertrophic scarring and significant relapse was seen in three and one patient, respectively, who underwent CO_2_ laser treatment.

Another study by Belmar treated 23 patients with a single session of CO_2_ laser. While 30% of patients’ angiofibromas were successfully treated with a single session, the majority had recurrence.

The use of CO_2_ laser for nodular type angiofibroma was first described by Biondo et al. The patient had extensive nodules that obstructed his nasal cavity and vision. The patient underwent two treatments with a three-month interval in between. The patient had some recurrence but at a significantly lesser rate.

CO_2_ laser ablation in treating angiofibromas has been modestly studied and reported in literature. All these studies addressed angiofibromas of the face. No specific treatment modalities have been reported regarding laser treatment for angiofibromas of the NAC. Given the sensitive nature of the NAC and the importance of preserving the NAC for future breast feeding as well as normal breast development, removal and treatment of the angiofibroma should be approached conservatively in order to preserve as much tissue as possible. While we did not notice recurrence at 8 months, our follow up period is short compared to those of studies mentioned above (up to 24 months). We will continue monitoring this patient and we will employ additional therapies as necessary.

Contraindications to CO_2_ laser therapy include bacterial, viral or fungal infection in the area to be treated [10]. People undergoing oral isotretinoin therapy should wait six months before starting CO_2_ laser therapy, although some surgeons prefer waiting up to 12 months [10]. Other relative contraindications include scarring following previous CO_2_ laser therapy, collagen vascular disease, immunosuppression, history of hypertrophic scar formation, history of autoimmune cutaneous diseases, and gold therapy [10].

## Conclusions

Cutaneous angiofibromas of the NAC are a rare occurrence and are often associated with TS. Due to the complexity of the NAC and the importance of the nipple in delivering breast milk, the NAC should be preserved, if possible. Utilizing a combined method of tangential debulking and CO_2_ laser ablation of the angiofibroma lesion properly removes the lesion while preserving the NAC.
